# Most small bowel cancers are revealed by a complication

**DOI:** 10.1590/S1679-45082015AO3380

**Published:** 2015

**Authors:** Ionut Negoi, Sorin Paun, Sorin Hostiuc, Bodgan Stoica, Ioan Tanase, Ruxandra Irina Negoi, Mircea Beuran

**Affiliations:** 1 Carol Davila University of Medicine and Pharmacy Bucharest, Bucharest, Romania.; 2 Emergency Hospital of Bucharest, Bucharest, Romania.

**Keywords:** Intestine, small/pathology, Neoplasms, Emergencies

## Abstract

**Objective:**

To characterize the pattern of primary small bowel cancers in a tertiary East-European hospital.

**Methods:**

A retrospective study of patients with small bowel cancers admitted to a tertiary emergency center, over the past 15 years.

**Results:**

There were 57 patients with small bowel cancer, representing 0.039% of admissions and 0.059% of laparotomies. There were 37 (64.9%) men, mean age of 58 years; and 72 years for females. Out of 57 patients, 48 (84.2%) were admitted due to an emergency situation: obstruction in 21 (38.9%), perforation in 17 (31.5%), upper gastrointestinal bleeding in 8 (14.8%), and lower gastrointestinal bleeding in 2 (3.7%). There were 10 (17.5%) duodenal tumors, 21 (36.8%) jejunal tumors and 26 (45.6%) ileal tumors. The most frequent neoplasms were gastrointestinal stromal tumor in 24 patients (42.1%), adenocarcinoma in 19 (33.3%), lymphoma in 8 (14%), and carcinoids in 2 (3.5%). The prevalence of duodenal adenocarcinoma was 14.55 times greater than that of the small bowel, and the prevalence of duodenal stromal tumors was 1.818 time greater than that of the small bowel. Obstruction was the complication in adenocarcinoma in 57.9% of cases, and perforation was the major local complication (47.8%) in stromal tumors.

**Conclusion:**

Primary small bowel cancers are usually diagnosed at advanced stages, and revealed by a local complication of the tumor. Their surgical management in emergency setting is associated to significant morbidity and mortality rates.

## INTRODUCTION

Malignant tumors of the small bowel are extremely rare, and account to, in average, approximately 2 to 3% of gastrointestinal cancers.^(1-3)^ The estimated new cases per year and the estimated mortality is 5,300 and 1,210, respectively, in the United States, and 3,500 and 1,100, respectively, in Europe.^(4)^ Their incidence increased during the last decades, and in roughly one third of cases they are associated with prior or subsequent other tumor of the gastrointestinal tract.^(5)^ More than 40 different histopathological types of neoplasm occur in the small bowel, but over 95% of malignancy are adenocarcinomas, gastrointestinal stromal tumors (GIST), carcinoids or lymphomas. The diagnosis of these tumors is often delayed due to their nonspecific symptoms, usually being made during an acute complication of the disease. In a symptomatic stage more than 50% of cases present metastatic disease.^(6)^ Knowing the nonspecificity of the clinical picture, only a high index of suspicion may offer an early diagnosis and treatment. The reported overall 5-year survival rate was 83% for carcinoids, 25% for adenocarcinomas, 62% for lymphomas and 45% for stromal tumors.^(7)^ However, the combined 5-year relative overall survival improved from 32.5 to 66.9%, between 1975 and 2006.^(8)^ Out of all the periampullary tumors, duodenal adenocarcinomas have the best prognosis, with a 5-year survival of 32.8%, comparing with pancreatic head adenocarcinoma which has a 5-year survival of only 6.5%.^(9)^ Overall, advanced small bowel adenocarcinoma had a worse prognosis than colorectal cancer, but better than gastric or pancreatic tumors.^(10)^

## OBJECTIVE

The main objective of this study was to characterize the pattern of small bowel primary cancers managed in a tertiary, university affiliated, emergency center. The secondary objectives were to determine early morbidity and mortality associated with surgical resection, and to correlate the pathology of the tumor with its clinical picture.

## METHODS

Retrospective study of adult patients, males and females, admitted in our hospital over the past 15 years. The selection criteria were duodenal, jejunal or ileal tumor; and histopathological examination proving the small bowel malignant neoplasm. Continuous variables are expressed as mean ± standard deviation, and the categorical ones as number (percent). Categorical variables were compared by the χ^(2)^tests. The distribution of continuous variables was evaluated by Kolmogorov-Smirnov test and one-way Analisys of Variance (ANOVA). The *t*-test or the nonparametric tests (Mann-Whitney U or Kruskal-Wallis H tests) were used for independent samples. The probability of rejecting the null hypothesis (statistical significance) was set at 0.05. For statistical analysis we used IBM Statistical Package for the Social Science (SPSS) Statistics 20 software. We also did an electronic search at the databases of the U.S. National Library of Medicine, National Institutes of Health, PubMed/MEDLINE, EMBASE, Google Scholar, and ISI Web of Knowledge, to identify original articles and reviews about the subject. The terms “intestinal”, “small bowel”, “cancer” and “tumor” were used in various combinations. The keywords were identified as truncated words in the title, abstract or in Medical Subject Heading (MeSH). Only English language literature was selected for further analysis. Electronic and manual cross-referencing was used further to find more relevant sources.

## RESULTS

**Figure 1:**
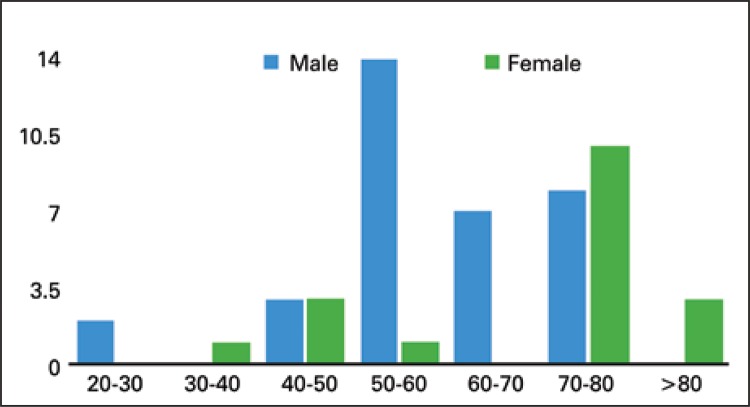
Age distribution of the patients

**Figure 2:**
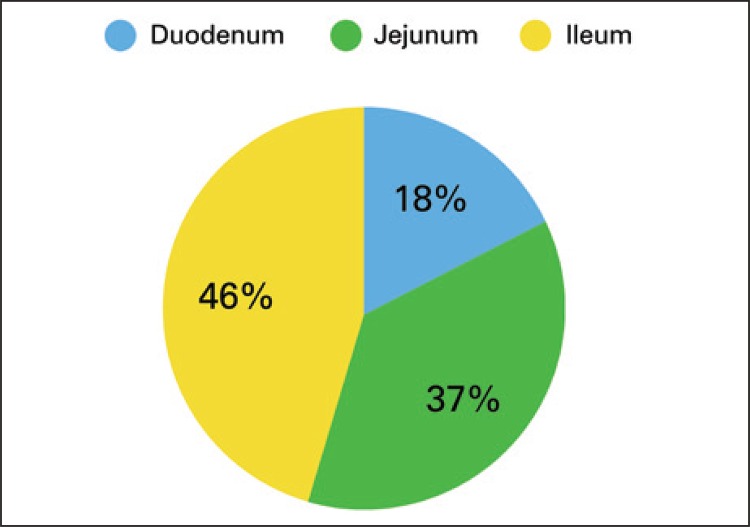
Localization of the small bowel tumor

There were 57 patients admitted during 15 years, accounting for 0.039% from our admissions and 0.059% from our laparotomies. There were 37 (64.9%) males and 20 (35.1%) females (95% confidence interval: 95%CI − female: 23%-48%; males: 52%-77%). The mean age for males was 58±12.7 years, and 68±16,20 years for females (Figure 1). There were 10 (17.5%) duodenal, 21 (36.8%) jejunal and 26 (45.6%) ileal tumors (Figure 2). Out of 57 patients, 48 (84.2%) were admitted in emergency setting: obstruction with 21 (38.9%) cases, perforation with 17 (31.5%), upper gastrointestinal bleeding with 8 (14.8%) and lower gastrointestinal bleeding with 2 (3.7%). We observed a mean interval from onset of symptoms to surgical treatment of 73 days, with 2.2±1.32 imaging tests per patient and 1.1±0.326 prior hospital admissions for nonspecific abdominal symptoms.

**Figure 3:**
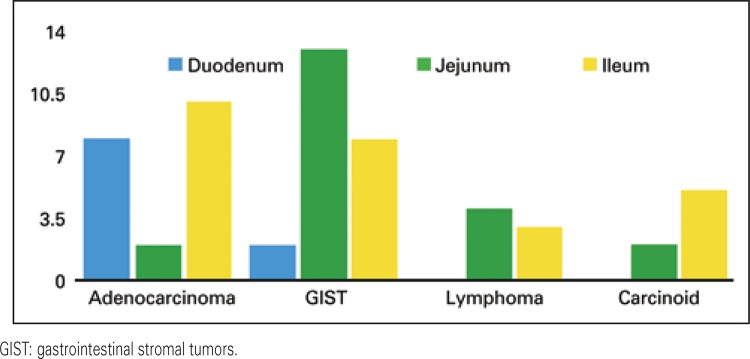
Histopathology of the tumor correlated with the location

**Figure 4:**
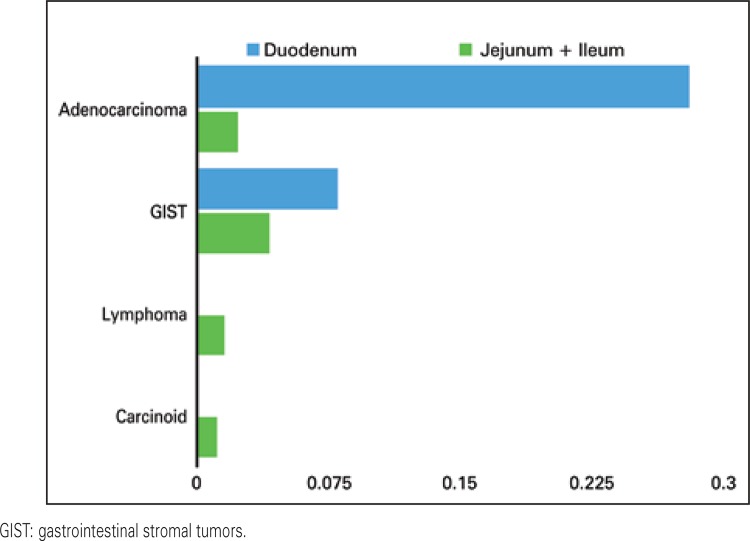
The observed frequency of the small bowel tumors correlated with the anatomical length of the duodenum *versus* jejunum and ileum

The most common conditions were GIST (24/42.1%), adenocarcinoma (19/33.3%), lymphoma (8/14%), and carcinoids (7/10.6%) (Figure 3). The prevalence of duodenal adenocarcinoma was 14.55 times greater than that of the small bowel (p=0.001), the prevalence of duodenal stromal tumors was 1.818 time greater than that of the small bowel (p=0.001) (Figure 4).

Intestinal obstruction was the complication observed in adenocarcinoma in 57.9% of cases (p=0.019), and tumor rupture was the main local complication (47.8%) for GIST (p=0.024). The surgical approach for duodenal tumors was pancreatoduodenectomy (PD) in 80%, tumorectomy in 10%, and palliative by-pass in 10% of cases. PD for complicated duodenal cancers strongly correlates with a high mortality (p=0.008). All cases of jejunal and ileal tumors were managed by intestinal resection with regional lymphadenectomy and primary anastomosis. We observed a 30-day mortality of 26%.

## DISCUSSION

Although the small bowel accounts for 75% of the length and 90% of the surface of the gastrointestinal tract, it very rarely presents malignancies. An explanation for this would be a more diluted content with less irritation of the mucosa, a rapid transit with a shorter exposure to the carcinogens, less bacterial flora with less conversion of the biliary acids into carcinogens, and an increased lymphoid tissue with a high local concentration of IgA, differently from the colorectal region.^(11,12)^ The gut has an area of 300m^(2)^, which is the largest barrier against the external environment, such as dietary components and the bacterial flora^(13)^ (Chart 1).

**Chart 1:** Predisposing conditions to small bowel cancer

Risk factors	Tumor type	
Small bowel adenoma^(14,15)^	Adenocarcinoma	Risk is more important for increased size, higher dysplasia grade and villous morphology
Crohn’s disease^(16)^	Adenocarcinoma	A relative risk of 33
Coeliac disease^(17,18)^	Adenocarcinoma and lymphoma	A relative risk for adenocarcinoma of 60-80
Hereditary non-polyposis colorectal cancer^(19)^	Adenocarcinoma	A relative risk more than 100
Familial adenomatous polyposis^(20)^	Adenocarcinoma	3-5% will develop duodenal cancer
Peutz Jeghers syndrome^(21)^	Adenocarcinoma	A relative risk of 520

The intestinal epithelium has an enormous self-renewing capacity, being completely replaced in 4 to 5 days.^(22)^ The carcinogenesis mechanisms seem to be related to the host-bacteria interaction, with secondary changes into the intestinal stem cell function, such as activation of the JAK-STAT, JNK and Wnt pathways. Chronic inflammation and hyperproliferation of the intestinal stem cells initiate malignant transformation, maintenance and metastases.^(23)^ Laforest et al. investigated the frequency of somatic mutations in 83 small bowel adenocarcinomas.^(24)^ These cases were selected from two European databases; in that, 47% of cases were duodenal tumors, and, in 63% of cases, there were three or more tumors. With a frequency over 5%, there were mutations in Kras*,* TP53, APC, SMAD4, PIK3CA, BRAF, ERBB2, BRAF and FBXW7. ERBB2 mutations were present in 12% of patients, and significantly associated with duodenal tumor location. This study suggests that more than 10% of patients with small bowel adenocarcinoma may benefit from anti-ERBB2-targeted agent.^(24)^

Maglinte et al. investigated the reasons for primary malignancies of the small bowel being diagnosed so late. They found that the average delay is attributable, as follows: (a) if less than 2 months, to patients failing to report symptoms; (b) at 8.2 months, to physicians not ordering the appropriate diagnostic tests; (c) at 12 months, to the radiologist failing to make diagnosis.^(25)^ We observed in our patients a mean interval from onset of symptoms to diagnosis of 73 days.

An analysis of the Surveillance, Epidemiology, and End Results (SEER), from 1973 to 1982, showed that the incidence of the malignant carcinoid increased during this decade by 50%.^(26)^ Carcinoid tumors, lymphomas, and sarcomas were rarely located in the duodenum, while nearly half of the adenocarcinomas were found in this site. A total of 87% of carcinoids and 60% of lymphomas were in the ileum.^(26)^ The analysis of 67,843 patients, from the National Cancer Data Database (1985-2005) and SEER (1973-2004) showed that 37.4% were carcinoids, 36.9% adenocarcinomas, 8.8% stromal tumors, and 17.3% lymphomas.^(27)^ We have found similarities for the frequency of adenocarcinoma (33.3%) and lymphoma (14%), with a significantly larger number of GIST (42.1%) and lesser for carcinoids (10.6%). These differences may be due to our hospital profile, being a tertiary emergency center, with carcinoids presenting a longer indolent clinical course and a lower complication rate comparing to GIST.

From 1974 to 2004, the incidence of carcinoids increased more than four-fold, from 2.1 to 9.3 per million, while changes in adenocarcinoma, GIST and lymphoma were less significant.^(27)^ A population-based study from Swedish Cancer Register analyzed 6,604 patients with small bowel malignant tumors.^(28)^ During the study period, from 1960-2009, the incidence of the duodenal cancer increased more than three-fold, especially by a dramatically rising trend of adenocarcinoma of the duodenum.^(28)^ In a study analyzing the data from Western Canada, most adenocarcinomas (54.7%) occurred in the duodenum, 29.9% in the jejunum and 16% in the ileum.^(29)^ There was an opposite trend for carcinoids, being located in only 3.9% of cases in duodenum, and in 9.2% in the jejunum and in 86.7% in ileum. The same disposition was for lymphomas: 21% in duodenum, 29.4% in jejunum and 49.5% in the ileum.^(29)^ Looking at the frequency of the tumors per centimeter of the small bowel, we observed an incidence, at the level of the duodenum, 14.55 times higher for adenocarcinomas and 1.818 higher for GIST.

An analysis of 1,060 patients from Connecticut Tumor Registry showed that the most common location was the ileum (29.7%), followed by duodenum (25.4%) and jejunum (15.3%). In 27.8% of cases the tumor location was not specified.^(5)^ We have found 17.5% duodenal tumors, 36.8% jejunal tumors and 45.6% ileal tumors.

Jejunal and ileal adenocarcinomas are best managed by wide segmental resection and regional lymphadenectomy. Right hemicolectomy is indicated for distal ileal tumor. Adenocarcinomas of the first and second duodenum should be addressed by PD, while tumors of the third and fourth duodenum through a pancreas sparing duodenal resection.^(8)^ For radical resections of the duodenal tumors we preferred PD in 80% of cases, while jejunal and ileal tumors were managed by segmental oncological resection with primary anastomosis in all cases.

Howe et al. reviewed the National Cancer Database, finding 4,995 patients with small bowel adenocarcinoma.^(30)^ The overall 5-year disease specific survival was 30.5%, with a median survival of 19.7 months. Factors significantly correlated with disease specific survival were age, tumor site, disease stage, and whether cancer directed surgery was performed. The disease specific survival is reduced for duodenal adenocarcinoma compared with jejunal or ileal tumors.^(30)^ Sohn et al. investigated factors influencing long-term survival in 55 patients with duodenal adenocarcinomas.^(31)^ From 48 patients with radical resections there were 35 PD and 13 pancreas-sparing duodenectomies. PD was associated with increased postoperative complications (57% *versus* 30%). The favorable factors for long-term survival were negative resection margins, PD and tumors in the first and second portions of the duodenum.^(31)^ The Cleveland Clinic group found the following negative prognostic factors for survival in adenocarcinoma of the small bowel: positive surgical margins, extramural venous spread, lymph node metastases, poor tumor differentiation, depth of tumor invasion, and history of Crohn’s disease.^(32)^ As an adjuvant chemotherapeutic regimen it seems that the combination of capecitabine and oxaliplatin is highly effective for small bowel adenocarcinoma, with a median overall survival favoring chemotherapy for advanced jejunal or ileal tumors of 17 *versus* 8 months (p=0.114).^(33)^ Czaykowski and Hui reviewed the 10-year experience of the British Columbia Cancer Agency regarding the chemotherapy effect in small bowel adenocarcinoma.^(34)^ The chemotherapy was given to 21 of the 47 patients. Out of 19 patients treated with curative intent, 5 received adjuvant chemotherapy. The median overall survival for those who received palliative chemotherapy was 15.6 months *versus* 7.7 months^(34)^ (Chart 2).

**Chart 2:** Studies evaluating chemotherapy in advanced small bowel adenocarcinoma

Reference	Regimen	Number of patients	Response rate (%)	Progression- free survival (months)	Overall survival (months)
Crawley et al.^(35)^	5FU	8	37	7.8	13.0
Locher et al.^(36)^	5FU + cisplatin	20	21	8.0	14.0
Gibson et al.^(37)^	5FU + doxorubicin + MMC	38	18	5.0	8.0
Zaanan et al.^(38)^	FOLFOX	48	34	6.9	17.8
	LV5FU2	10	0	7.7	13.5
	LV5FU2 +cisplatin	19	30	6.0	9.6
	FOLFIRI	16	9	4.8	10.6
Overman et al.^(39)^	5FU + cisplatin	29	41	8.7	14.8
	5FU without cisplatin	41	17	3.9	12.0
Overman et al.^(40)^	Capecitabine + oxaliplatin	30	52	11.3	20.0
Zaanan et al.^(41)^	FOLFIRI (second line)	28	20	3.2	10.5

5FU: 5-fluorouracil; MMC: mitomycin C; FOLFOX: 5-fluorouracil/leucovorin/oxaliplatin; LV5FU2: 5-fluorouracil/leucovorin; FOLFIRI: 5-fluorouracil/leucovorin/irinotecan. Reprinted from: Aparicio et al.,^(10)^ with permission from Elsevier.

Carcinoids are neuroendocrine tumors and upon diagnosis, 29% of cases are at a localized stage, 41% in a loco-regional stage, and 30% of patients present metastates.^(42)^ Of patients with midgut carcinoid tumors 40% have a second gastrointestinal neoplasm, which requires a careful diagnostic evaluation prior to surgery of these tumors.^(43)^ The rate of lymph node and distant metastases is 12 and 5%, respectively, in tumor smaller than 1cm, and 85 and 47%, respectively, for tumor greater than 2cm.^(8)^ Surgery represents the main approach for localized disease, with wide excision of the bowel and mesentery. Peritoneal carcinomatosis may be present in up to 30% of patients with small intestine primary neuroendocrine tumors.^(44)^ For carefully selected patients presenting neuroendocrine tumors-related peritoneal carcinomatosis, it seems that cytoreductive surgery prolongs survival, especially when peritoneal lesions are completed resected.^(44)^

## CONCLUSION

Primary small bowel cancers are usually diagnosed at an advanced stage, and revealed by a local complication of the tumor. Their surgical approach in emergency setting carries specific morbidity and significant mortality, but only a standard radical R0 resection with regional lymph node dissection may provide the patients the best chance for cure.
