# Supplementary material for: Most small bowel cancers are revealed by a complication
Source: Einstein (Sao Paulo). 2015 Oct-Dec;13(4):500–5. doi: 10.1590/S1679-45082015AO3380 (PMC4878621; doi:10.1590/S1679-45082015AO3380)
Supplement: Translation

# Translation: A maioria dos cânceres de intestino delgado são revelados por uma complicação

**Authors:** Ionut Negoi, Sorin Paun, Sorin Hostiuc, Bodgan Stoica, Ioan Tanase, Ruxandra Irina Negoi, Mircea Beuran

**Affiliations:** 1 Carol Davila University of Medicine and Pharmacy Bucharest, Bucareste, Romênia.; 2 Emergency Hospital of Bucharest, Bucareste, Romênia.

**Keywords:** Intestino delgado/patologia, Neoplasias, Emergências

## Abstract

**Objetivo:**

Caracterizar o padrão de neoplasias malignas primárias do intestino delgado em um hospital terciário de Leste Europeu.

**Métodos:**

Estudo retrospectivo de pacientes com câncer de intestino delgado, internados em um hospital terciário e de emergência, ao longo dos últimos 15 anos.

**Resultados:**

Foram avaliados 57 pacientes com neoplasias malignas gastrintestinais, o que representou 0,039% das admissões e 0,059% das laparotomias realizadas. Total de 37 (64,9%) pacientes masculinos, média de idade de 58 anos, e de 72 anos para mulheres. Dentre os 57 pacientes, 48 (84,2%) foram internados em situação de emergência: obstrução intestinal em 21 (38,9%), perfuração em 17 (31,5%), hemorragia digestiva alta em 8 (14,8%), e hemorragia digestiva baixa em 2 (3,7%). Houve 10 (17,5%) tumores duodenais, 21 (36,8%) jejunais e 26 (45,6%) ileais. As neoplasias mais frequentes foram tumor estromal gastrintestinal, em 24 (42,1%) pacientes, adenocarcinoma em 19 (33,3%), linfoma em 8 (14%) e carcinoides em 2 (3,5%). A prevalência de adenocarcinoma duodenal foi 14,55 vezes maior do que a do intestino delgado, e a prevalência de tumores estromais duodenais foi 1,818 vez maior do que a do intestino delgado. A obstrução intestinal foi complicação do adenocarcinoma em 57,9% dos casos, e a perfuração foi a principal complicação local (47,8%) dos tumores estromais.

**Conclusão:**

As neoplasias malignas primárias do intestino delgado foram geralmente diagnosticadas em estado avançado e reveladas por uma complicação local do tumor. O tratamento cirúrgico em situação de emergência está associado à significativa morbimortalidade.

## INTRODUÇÃO

As neoplasias malignas do intestino delgado são extremamente raras e representam, em média, aproximadamente 2 a 3% das neoplasias gastrintestinais.^(1-3)^ Os novos casos estimados por ano e a mortalidade estimada são de 5.300 e 1.210, respectivamente, nos Estados Unidos, e de 3.500 e 1.100, respectivamente, na Europa.^(4)^ Sua incidência aumentou nas últimas décadas e, em cerca de um terço dos casos, esteja associada a outro tumor do trato gastrintestinal.^(5)^ Mais de 40 diferentes tipos histopatológicos de neoplasia ocorrem no intestino delgado, porém mais de 95% dos casos malignos são adenocarcinomas, tumores estromais gastrintestinais (GIST - *gastrointestinal stromal tumors*), carcinoides ou linfomas. O diagnóstico desses tumores muitas vezes é tardio, devido a sintomas inespecíficos, ocorrendo normalmente em uma complicação aguda da doença. Em um estágio sintomático, mais de 50% dos casos apresentam doença metastática.^(6)^ Conhecendo a não especificidade do quadro clínico, somente um alto índice de suspeita pode oferecer diagnóstico e tratamento precoces. A taxa relatada de sobrevida global em 5 anos foi de 83% para os carcinoides, 25% para os adenocarcinomas, 62% para os linfomas e 45% para os tumores estromais.^(7)^ No entanto, a sobrevida global combinada em 5 anos melhorou entre 1975 e 2006, subindo de 32,5 para 66,9%.^(8)^ De todos os tumores periampulares, os adenocarcinomas duodenais têm o melhor prognóstico, com sobrevida em 5 anos de 32,8%, quando comparados aos adenocarcinomas da cabeça do pâncreas, que têm uma sobrevida em 5 anos de apenas 6,5%.^(9)^ De forma geral, o adenocarcinoma avançado do intestino delgado teve um prognóstico pior do que a neoplasia colorretal, mas melhor do que os tumores gástricos ou pancreáticos.^(10)^

## OBJETIVO

O principal objetivo deste estudo foi caracterizar o padrão de neoplasias primárias do intestino delgado em um centro universitário terciário de emergência. Os objetivos secundários foram determinar a morbimortalidade precoce associada à ressecção cirúrgica e correlacionar a patologia tumoral a seu quadro clínico.

## MÉTODOS

Estudo retrospectivo de pacientes adultos, dos sexos masculino e feminino, admitidos em nosso hospital nos últimos 15 anos. Os critérios de seleção foram tumor duodenal, jejunal ou ileal; e exame histopatológico comprovando a neoplasia maligna de intestino delgado. As variáveis contínuas são expressas na forma de média ± desvio padrão, e as categóricas na forma de número (porcentagem). As variáveis categóricas foram comparadas por meio de testes χ^(2)^. A distribuição de variáveis contínuas foi avaliada pelo teste de Kolmogorov-Smirnov e por Análise de Variância (ANOVA). O teste *t* ou seus similares não paramétricos (testes Mann-Whitney U ou Kruskal-Wallis H) foram usados para amostras independentes. A probabilidade de rejeição da hipótese nula (significância estatística) foi estabelecida em 0,05. Para a análise estatística, utilizamos o *software* IBM *Statistical Package for the Social Science* (SPSS) *Statistics* 20. Também fizemos uma busca eletrônica nas bases de dados *U.S. National Library of Medicine*, *National Institutes of Health*, PubMed/MEDLINE, EMBASE, Google Scholar e ISI *Web of Knowledge* para identificar artigos originais e revisões sobre o assunto. Os termos “intestinal”, “intestino delgado”, “neoplasia” e “tumor” foram utilizados em várias combinações. As palavras-chave foram identificadas como palavras truncadas no título, no resumo ou no vocabulário controlado *Medical Subject Heading* (MeSH). Somente a literatura em língua inglesa foi selecionada para uma análise mais detalhada. Referências cruzadas eletrônicas e manuais também foram utilizadas para encontrar fontes mais relevantes.

## RESULTADOS

Foram avaliados 57 pacientes internados em um período de 15 anos, representando 0,039% de nossas internações e 0,059% de nossas laparotomias. Havia 37 pacientes do sexo masculino (64,9%) e 20 (35,1%) do sexo feminino (intervalo de confiança de 95%: IC95% − sexo feminino: 23%-48%; sexo masculino: 52%-77%). A média de idade para os pacientes do sexo masculino foi 58±12,7 anos, e 68±16,20 anos para o feminino (Figura 1). Houve 10 (17,5%) tumores duodenais, 21 (36,8%) jejunais e 26 (45,6%) ileais (Figura 2). Dentre os 57 pacientes, 48 (84,2%) foram internados em situação de emergência: obstrução em 21 casos (38,9%), perfuração em 17 (31,5%), hemorragia digestiva alta em 8 (14,8%) e sangramento gastrintestinal pouco intenso em 2 (3,7%). Observamos um intervalo médio de 73 dias do início dos sintomas até o tratamento cirúrgico, com 2,2±1,32 testes de imagem por paciente e 1,1±0,326 admissões hospitalares prévias para sintomas abdominais não específicos.

**Figura 1:** Distribuição dos pacientes por idade

**Figura 2:** Localização do tumor no intestino delgado

As patologias mais comuns foram os GIST (24/42,1%), adenocarcinomas (19/33,3%), linfomas (8/14%) e carcinoides (7/10,6%) (Figura 3). A prevalência do adenocarcinoma duodenal foi 14,55 vezes maior do que a do adenocarcinoma do intestino delgado (p=0,001), e a prevalência de tumores estromais duodenais foi 1,818 vez maior do que a de tumores do intestino delgado (p=0,001) (Figura 4).

**Figura 3:** Histopatologia do tumor correlacionada à localização

**Figura 4:** Frequência observada de tumores do intestino delgado correlacionada ao comprimento anatômico do duodeno *versus* jejuno e íleo

A obstrução intestinal foi complicação do adenocarcinoma em 57,9% dos casos (p=0,019), e a ruptura tumoral foi a principal complicação local (47,8%) dos GIST (p=0,024). A abordagem cirúrgica dos tumores duodenais foi a pancreatoduodenectomia (PD) em 80% dos casos, a tumorectomia em 10%, e uma derivação paliativa em 10% dos casos. A PD para neoplasias duodenais complicadas se correlacionou fortemente a uma alta mortalidade (p=0,008). Todos os casos de tumores jejunais e ileais foram tratados por meio de ressecção intestinal com linfadenectomia e anastomose primária. Observamos uma mortalidade em 30 dias de 26%.

## DISCUSSÃO

Apesar do intestino delgado representar 75% da extensão e 90% da superfície do trato gastrintestinal, malignidades nesse local são muito raras. Uma explicação para isso seria um conteúdo mais diluído com menos irritação da mucosa, um trânsito rápido com menor exposição aos carcinogênicos, uma flora bacteriana bem menor com menos conversão dos ácidos biliares em carcinogênicos, e um tecido linfoide aumentado com uma alta concentração local de IgA.^(11,12)^ O intestino tem uma área de 300m^(2)^, que representa a maior barreira contra o ambiente externo, como os componentes da dieta e a flora bacteriana^(13)^ (Quadro 1).

**Quadro 1:** Doenças que predispõem ao câncer do intestino delgado

Fatores de risco	Tipo de tumor	
Adenoma do intestino delgado^(14,15)^	Adenocarcinoma	Risco mais importante para maior tamanho, maior grau de displasia e morfologia de vilosidades
Doença de Crohn^(16)^	Adenocarcinoma	Risco relativo de 33
Doença celíaca^(17,18)^	Adenocarcinoma e linfoma	Risco relativo para adenocarcinoma de 60-80
Câncer colorretal hereditário não polipose^(19)^	Adenocarcinoma	Risco relativo maior do que 100
Polipose adenomatosa familiar^(20)^	Adenocarcinoma	3 a 5% irão desenvolver câncer duodenal
Síndrome de Peutz Jeghers^(21)^	Adenocarcinoma	Risco relativo de 520

O epitélio intestinal tem uma enorme capacidade de autorrenovação, sendo completamente substituído em 4 a 5 dias.^(22)^ Os mecanismos carcinogênicos parecem estar relacionados à interação hospedeiro-bactéria, com mudanças secundárias na função das células-tronco intestinais, como a ativação das vias JAK-STAT, JNK e Wnt. A inflamação crônica e a hiperproliferação das células-tronco intestinais iniciam a transformação maligna, manutenção e metástases.^(23)^ Laforest et al. investigaram a frequência das mutações somáticas em 83 adenocarcinomas de intestino delgado.^(24)^ Esses casos foram selecionados a partir de duas bases de dados europeias; sendo 47% tumores duodenais e, em 63% dos casos, havia 3 ou mais tumores. Com uma frequência superior a 5%, houve mutações de Kras*, *TP53, APC, SMAD4, PIK3CA, BRAF, ERBB2, BRAF e FBXW7*. *As mutações de ERBB2 estavam presentes em 12% dos pacientes e significativamente associadas à localização do tumor duodenal. Esse estudo sugere que mais de 10% dos pacientes com adenocarcinoma de intestino delgado podem se beneficiar de um agente anti-ERBB2 direcionado.^(24)^

Maglinte et al. investigaram o motivo do diagnóstico das neoplasias primárias do intestino delgado ser feito tão tardiamente. Descobriram que o atraso médio pode ser explicado, como a seguir: (a) quando inferior a 2 meses, pelo fato de os pacientes não relatarem os sintomas; (b) com 8,2 meses, pelos médicos não solicitarem os exames diagnósticos adequados; (c) com 12 meses, pela falta de diagnóstico por parte do radiologista.^(25)^ Observamos, em nossos pacientes, um intervalo médio de 73 dias do início dos sintomas até o diagnóstico.

Uma análise do *Surveillance, Epidemiology, and End Results* (SEER), de 1973 a 1982, mostrou que a incidência do carcinoide maligno aumentou em 50% durante essa década.^(26)^ Tumores carcinoides, linfomas e sarcomas raramente estiveram localizados no duodeno, enquanto quase metade dos adenocarcinomas foi encontrada nesse local. O íleo apresentou 87% dos carcinoides e 60% dos linfomas.^(26)^ A análise de 67.843 pacientes feita pelo *National Cancer Data Database *(1985-2005) e pelo SEER (1973-2004) mostrou que 37,4% eram carcinoides, 36,9% adenocarcinomas, 8,8% tumores estromais e 17,3% linfomas.^(27)^ Encontramos semelhanças com relação à frequência de adenocarcinomas (33,3%) e de linfomas (14%), com um número significativamente maior de GIST (42,1%) e menor de carcinoides (10,6%). Essas diferenças podem ser explicadas pelo perfil do nosso hospital, que é um centro terciário de emergência, com os carcinoides apresentando um curso clínico indolor mais longo e uma taxa de complicações menor quando comparados aos GIST.

De 1974 a 2004, a incidência de carcinoides aumentou mais de quatro vezes, de 2,1 para 9,3 por milhão, enquanto as mudanças no adenocarcinoma, GIST e linfoma foram menos significativas.^(27)^ Um estudo de base populacional do *Swedish Cancer Register* analisou 6.604 pacientes com tumores malignos do intestino delgado.^(28)^ Durante o período de estudo, de 1960 a 2009, a incidência de neoplasia duodenal aumentou mais do que três vezes, especialmente por uma tendência de elevação radical do adenocarcinoma do duodeno.^(28)^ Em um estudo que analisou dados da região oeste do Canadá, a maioria dos adenocarcinomas (54,7%) ocorreu no duodeno, 29,9% no jejuno e 16% no íleo.^(29)^ Houve uma tendência oposta para os carcinoides, localizados no duodeno em somente 3,9% dos casos, 9,2% no jejuno e 86,7% no íleo. A mesma distribuição foi encontrada para os linfomas: 21% no duodeno, 29,4% no jejuno e 49,5% no íleo.^(29)^ Analisando a frequência dos tumores por centímetro do intestino delgado, observamos uma incidência 14,55 vezes maior de adenocarcinomas e 1,818 vez maior de GIST no nível do duodeno.

Uma análise de 1.060 pacientes do *Connecticut Tumor Registry* mostrou que a localização mais comum foi no íleo (29,7%), seguida pelo duodeno (25,4%) e jejuno (15,3%). Em 27,8% dos casos, a localização do tumor não foi definida.^(5)^ Encontramos 17,5% de tumores duodenais, 36,8% de tumores jejunais e 45,6% de tumores ileais.

Os adenocarcinomas jejunais e ileais são melhor tratados por meio de uma ressecção segmentar ampla e uma linfadenectomia regional. A hemicolectomia direita é indicada para o tumor ileal distal. Os adenocarcinomas do duodeno superior e ascendente devem ser tratados por meio de PD, enquanto os tumores do duodeno inferior e ascendente devem ser tratados com uma ressecção duodenal com preservação do pâncreas.^(8)^ Para ressecções radicais dos tumores duodenais, preferimos a PD em 80% dos casos, enquanto os tumores jejunais e ileais foram tratados com uma ressecção oncológica segmentar com anastomose primária em todos os casos.

Howe et al. analisaram a *National Cancer Database* e encontraram 4.995 pacientes com adenocarcinoma do intestino delgado.^(30)^ A sobrevida específica para a doença em 5 anos foi de 30,5%, com sobrevida média de 19,7 meses. Os seguintes fatores apresentaram correlação significativa com a sobrevida específica para a doença: idade, local do tumor, estágio da doença, e se uma cirurgia direcionada para a neoplasia foi realizada. A sobrevida específica para a doença é reduzida no adenocarcinoma duodenal quando comparada aos tumores jejunais ou ileais.^(30)^ Sohn et al. investigaram fatores que influenciam na sobrevida de longo prazo em 55 pacientes com adenocarcinomas duodenais.^(31)^ Dentre 48 pacientes com ressecções radicais, houve 35 PD e 13 duodenectomias com preservação do pâncreas. A PD esteve associada a um aumento das complicações pós-operatórias (57% *versus* 30%). Os fatores favoráveis à sobrevida em longo prazo foram margens de ressecção negativas, PD e tumores na primeira e segunda porções do duodeno.^(31)^ O grupo da *Cleveland Clinic* encontrou os seguintes fatores prognósticos negativos para a sobrevida no adenocarcinoma do intestino delgado: margens cirúrgicas positivas, propagação venosa extramural, metástases em linfonodos, pouca diferenciação tumoral, profundidade da invasão tumoral, e história de doença de Crohn.^(32)^ Como regime quimioterápico adjuvante, uma combinação de capecitabina e oxaliplatina é aparentemente altamente eficiente para o adenocarcinoma de intestino delgado, com uma mediana de sobrevida global de 17 *versus* 8 meses (p=0,114), favorecendo a quimioterapia para tumores jejunais ou ileais avançados.^(33)^ Czaykowski e Hui revisaram a experiência de 10 anos da *British Columbia Cancer Agency* sobre o efeito da quimioterapia no adenocarcinoma de intestino delgado.^(34)^ O tratamento quimioterápico foi administrado em 21 dos 47 pacientes. Dos 19 pacientes tratados com intenção curativa, 5 receberam quimioterapia adjuvante. A mediana de sobrevida global para aqueles que receberam quimioterapia paliativa foi 15,6 meses *versus* 7,7 meses^(34)^ (Quadro 2).

**Quadro 2:** Estudos que avaliaram quimioterapia em adenocarcinoma avançado do intestino delgado

Referência	Regime	Número de pacientes	Taxa de resposta (%)	Sobrevida livre de progressão (meses)	Sobrevida global (meses)
Crawley et al.^(35)^	5FU	8	37	7,8	13,0
Locher et al.^(36)^	5FU + cisplatina	20	21	8,0	14,0
Gibson et al.^(37)^	5FU + doxorubicina + MMC	38	18	5,0	8,0
Zaanan et al.^(38)^	FOLFOX	48	34	6,9	17,8
	LV5FU2	10	0	7,7	13,5
	LV5FU2 +cisplatina	19	30	6,0	9,6
	FOLFIRI	16	9	4,8	10,6
Overman et al.^(39)^	5FU + cisplatina	29	41	8,7	14,8
	5FU sem cisplatina	41	17	3,9	12,0
Overman et al.^(40)^	Capecitabina + oxaliplatina	30	52	11,3	20,0
Zaanan et al.^(41)^	FOLFIRI (segunda linha)	28	20	3,2	10,5

5FU: 5-fluorouracil; MMC: mitomicina C; FOLFOX: 5-fluorouracil/leucovorin/ oxaliplatina; LV5FU2: 5-fluorouracil/leucovorin; FOLFIRI: 5-fluorouracil /leucovorin/irinotecan. Reimpresso a partir de: Aparicio et al.,^(10)^ com permissão da Elsevier.

Os carcinoides são tumores neuroendócrinos e, à época do diagnóstico, 29% encontram-se em um estágio localizado, 41% em estágio loco-regional, e 30% em um estágio metastático.^(42)^ Dentre os pacientes com tumores carcinoides do intestino médio, 40% têm uma segunda neoplasia gastrintestinal, sendo necessária uma avaliação diagnóstica cuidadosa antes da cirurgia desses tumores.^(43)^ A taxa de metástases de linfonodos e metástases distantes é de 12 e 5%, respectivamente, em tumores menores do que 1cm, e de 85 e 47%, respectivamente, para tumores maiores do que 2cm.^(8)^ A cirurgia representa a principal abordagem para a doença localizada, com a excisão ampla do intestino e do mesentério. A carcinomatose peritoneal pode estar presente em até 30% dos pacientes com tumores neuroendócrinos primários do intestino delgado.^(44)^ Para os pacientes com carcinomatose peritoneal relacionada aos neuroendócrinos primários do intestino delgado, cuidadosamente selecionados, a cirurgia citorredutiva prolonga a sobrevida, em especial quando as lesões peritoneais são ressecadas por completo.^(44)^

## CONCLUSÃO

As neoplasias malignas primárias do intestino delgado são geralmente diagnosticadas em estágio avançado e reveladas por uma complicação local do tumor. Sua abordagem cirúrgica na situação de emergência traz uma morbidade específica e uma mortalidade significativa, mas somente uma ressecção R0 radical padrão, com dissecção de linfonodos regionais pode oferecer aos pacientes a melhor chance de cura.
